# The Relationship Between Mindfulness and Learning Burnout Among High School Students: The Chain-Mediating Role of Future Time Perspective and Academic Positive Emotions

**DOI:** 10.3390/bs16020188

**Published:** 2026-01-28

**Authors:** Wenmei Sun, Qianqian Niu, Xubo Liu

**Affiliations:** Faculty of Education, Henan Normal University, Xinxiang 453007, China; 2310214002@stu.htu.edu.cn (Q.N.); 19100274046@stu.htu.edu.cn (X.L.)

**Keywords:** mindfulness, future time perspective, academic positive emotions, learning burnout

## Abstract

Grounded in the self-regulated learning model, this research examined the effects of mindfulness on learning burnout among high school students. A survey was administered to 1311 high school students utilizing the following instruments: the Short Form of the Five Facet Mindfulness Questionnaire, the Future Time Perspective Scale, the Academic Positive Emotions Questionnaire, and the Learning Burnout Scale for Middle School Students. The outcomes implied that (1) mindfulness exhibited an inverse association with learning burnout in high school students, (2) indirect effects consistent with the independent mediating roles of future time perspective and academic positive emotions on learning burnout, and (3) exerted an indirect effect on learning burnout via the sequential mediation of future time perspective and subsequent academic positive emotions. Mindfulness can alleviate learning burnout and promote the physical and mental health of high school students by enhancing future time perspective and academic positive emotions.

## 1. Introduction

The self-regulated learning (SRL) model posits that self-regulated learning refers to a psychological process in which learners actively participate in the learning process, autonomously monitoring and managing their progress to achieve learning goals—a process of self-management and self-organization (Edisherashvili et al., 2022; Jia et al., 2024; Zimmerman & Schunk, 2001). Within this framework, learners’ meta-cognition, motivation-affect, and behavioral strategies are crucial for their academic adaptation (Jia et al., 2024; J. K. Wang et al., 2023). However, despite the continuous advancement of the Ministry of Education’s “Double Reduction” policy, which has alleviated the academic burden on Chinese adolescents (Zhou & Qi, 2022), data from multiple surveys indicate that learning burnout (LB)—a prominent state of maladaptive academic adjustment—remains prevalent and severe among high school students, harming their physical and mental health and thus requiring urgent resolution (W. Chen et al., 2016; Ji et al., 2020). LB manifests as students’ feelings of exhaustion and detachment, characterized by diminished enthusiasm for learning, physical and mental depletion, and a cynical attitude toward educational tasks (Cao, 2018; Hu & Dai, 2007). Multiple empirical studies suggest that LB is often triggered by a combination of academic pressure and individual susceptibility (Gao, 2023; Ji et al., 2020; M. Jin et al., 2022; Zhu & Chang, 2021). It not only negatively associated with students’ academic cognition and achievement (Dong & Yu, 2010), but also has extensive adverse effects on their physiological, psychological, behavioral, and interpersonal functioning (Bilige & Gan, 2020; M. T. Wang et al., 2015). LB has become one of key indicators of student mental health (He et al., 2019). Exploring the factors that influence LB and its internal mechanisms is a topic of close concern in basic education.

Self-regulated learning (SRL) exerts a notable inverse predictive effect on their LB (Barbosa et al., 2016; Poorgholamy et al., 2020; Z. Wang & Zheng, 2023) and serves as a decisive factor for learners to successfully implement the learning process and improve their academic performance (Broadbent & Poon, 2015; Veenman, 2011; Wong et al., 2019). As a meta-cognitive component grounded in self-regulated learning, mindfulness has been demonstrated to play a role in reducing LB (Calvete et al., 2017; Tao, 2017). However, an in-depth exploration of the relationship between mindfulness and LB is still lacking. Mindfulness refers to an individual’s conscious focus on attention, non-judgmental concentration on internal experiences, bodily sensations, and the state of present-moment events—an active psychological trait (Kabat-Zinn, 2003; Bishop et al., 2004). The Self-Determination Theory suggests that mindfulness, as an important internal personal resource, helps individuals focus their attention on meeting current demands, enhancing their sense of control, and satisfying their needs for autonomy and competence (Y. Li et al., 2021). In fact, LB stems from inefficacy or an “efficacy crisis” and a key element of LB is the lack of confidence in one’s abilities (Cherniss, 1993). Therefore, mindfulness can compensate for the unmet psychological needs caused by LB and prevent the resulting “efficacy crisis.” Moyers (1993) noted that nearly all individuals can benefit from enhanced awareness. Mindfulness, as a heightened awareness of the self, enables individuals to perceive their learning processes more holistically and access more positive psychological experiences, thereby reducing the likelihood of LB. Previous studies have also indicated that even brief mindfulness practice can induce relaxation, thus diminishing the potential for LB (Creswell, 2017). In essence, by enhancing an individual’s awareness of present experiences, capacity for concentration, and emotion regulation ability, mindfulness can amplify feelings of immersion and pleasure. This, in turn, facilitates psychological recovery and relaxation, ultimately alleviating LB. Furthermore, by fostering an open and accepting attitude toward personal experiences, mindfulness can help individuals notice and regulate the maladaptive thoughts and emotional responses, which helps them overcome LB. To sum up, Hypothesis 1 (H1) was proposed: mindfulness would demonstrate a significant negative correlation with LB among the high school student population.

Future Time Perspective (FTP) refers to an individual’s cognitive representation and positive expectation toward their personal future. Characterized as a key personality disposition, FTP is closely associated with planning proficiency. Its core function is to facilitate goal setting and prospective planning (Carstensen, 2006; Z. Jin, 2011; W. Zhang & Wood, 2025), which simultaneously promotes individual development (X. Huang, 2004). Relevant studies indicate that FTP demonstrates a statistically marked positive association with learning engagement; individuals with higher FTP exhibit better learning engagement states (Qu, 2023). Furthermore, according to Goal Setting Theory, clear goals are central to directing and sustaining behavior (Locke & Latham, 2002). A deficit in FTP signifies the vagueness or loss of long-term goal orientation (Bandura, 2001), which can easily lead to a lack of focus in learning behaviors and a diminished sense of accomplishment-core characteristics of LB (G. Song et al., 2013). Consequently, a lack of FTP may contribute to the emergence of LB among high school students. Conversely, by highlighting the utility of present behaviors for shaping future outcomes, positive FTP motivates students to reinforce their academic goals, initiate more effective learning behaviors, and decrease LB (Pang et al., 2014). The balanced time perspective theory posits that individuals possess three interrelated time perspectives: past, present, and future (Zimbardo & Boyd, 1999). Mindfulness and FTP represent positive present and future perspectives, respectively, and can potentially influence each other (Kabat-Zinn, 2003). Mindfulness embodies present-moment awareness and intentional attention. Elevated mindfulness levels correlate with a constructive stance toward the present. Consequently, they are inclined to perceive the future as adaptable and promising rather than fatalistic, thereby fostering a more positive future time perspective (Zimbardo & Boyd, 2008). Previous research has also indicated that a positive present-time perspective positively predicts positive FTP (Rönnlund et al., 2019). Mindfulness can significantly and positively predict FTP (Kabat-Zinn, 2003). In summary, Hypothesis 2 (H2) was proposed: mindfulness can reduce LB through FTP.

Academic positive emotions (APE) are defined as a range of positive emotional experiences pertinent to students’ academic activities in the context of teaching or learning (Dong & Yu, 2007). According to the broaden-and-build theory (BBT) of positive emotions, the capacity of positive emotions to broaden momentary thought-action repertoires and build enduring personal resources serves to reduce emotional exhaustion and mitigate LB (Fredrickson, 2001). Prior studies have further demonstrated that APE broaden individuals’ cognitive and behavioral resources, thereby enhancing academic performance (L. Li et al., 2020; Pekrun et al., 2017) and alleviating LB. Similarly, the academic emotions theory posits that APE significantly positively impact individuals’ self-regulated learning, meaning that they promote self-regulated learning, thereby reducing LB (Pekrun et al., 2002). Meta-analysis results on academic emotions also indicate that APE in high school students promote learning outcomes and reduce LB (Tan et al., 2021). Individuals with high mindfulness levels perceive the world more authentically, possess a more positive present-time perspective, and consequently experience more positive emotions. Existing research confirms that mindfulness, as a psychological protective factor, can effectively improve attentional functions, direct and sustain most attention toward behavioral goals, and increase tolerance and acceptance of negative emotions, thereby helping individuals deeply experience the pleasure derived from positive life events (Biegel et al., 2009; Goldin & Gross, 2010). Meanwhile, mindfulness directly assists students in maintaining APE, thereby boosting their positive affect and life satisfaction. This, in turn, mitigates LB (Calvete et al., 2017; Dong & Yu, 2010; M. Jin et al., 2022). Concurrently, mindfulness-intervention research has proven that mindfulness training strengthens positive emotional experiences and reduces LB by enhancing individual mindfulness levels, with these positive effects lasting for at least ten months (Kinnunen et al., 2020). In summary, Hypothesis 3 (H3) was proposed: mindfulness can reduce LB by increasing APE.

**Figure 1:**
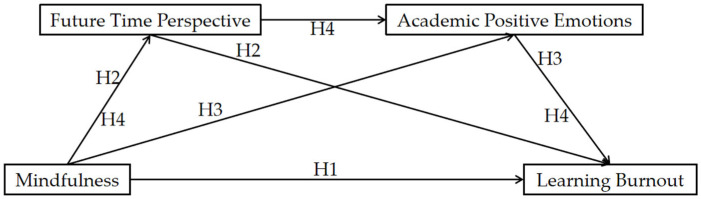
Hypothesized model.

The self-regulated learning model posits that self-regulated learners essentially control their own learning, with active guidance and mastery of cognitive and motivational processes being the most salient features (Boekaerts & Cascallar, 2006). Within this model, three components—meta-cognition, cognition, and motivation-affect—form a hierarchical and progressive relationship (Edisherashvili et al., 2022). Specifically, meta-cognition influences cognition, which in turn affects motivation-affect, ultimately leading to changes in individual behavior. In the meantime, previous research has further demonstrated that the motivational-affective component of self-regulated learning model is persistently influenced to a large extent by factors associated with the success or failure of the meta-cognitive and cognitive components (Edisherashvili et al., 2022). When an individual’s meta-cognitive ability strengthens, their learning cognition changes, subsequently altering motivational-affective aspects to improve learners’ behaviors and attitudes toward learning. Meta-cognition refers to an individual’s ability to cognize, monitor, and regulate their own cognitive processes, inherently influencing cognitive processes. Mindfulness emphasizes focusing attention on present-moment awareness, belonging to the meta-cognitive component of self-regulated learning; FTP involves cognition of future time, which belongs to the cognitive component of self-regulated learning; APE belongs to the motivational-affective component of self-regulated learning. Moreover, empirical evidence indicates that FTP serves as a catalyst for the development of APE (Q. Huang & Zhang, 2022). Individuals with high mindfulness levels pay more attention to their current state, hold more positive views about the future, possess stronger FTP, experience richer and more frequent APE during learning, exhibit stronger learning motivation and engagement, and demonstrate a marked reduction in LB. Thus, Hypothesis 4 (H4) was proposed: FTP and APE act as sequential mediators linking mindfulness to LB. The model is illustrated in Figure 1.

Drawing on the self-regulated learning model, this study investigated how mindfulness influences LB in high school students, along with mediating mechanism involving FTP and APE. This research aimed to establish a theoretical and practical foundation for mitigating LB and promoting academic, psychological, and physical development among high school students.

## 2. Method

### 2.1. Participants

Data were collected from a high school in a Chinese city using a convenience sample method. Out of 1345 questionnaires that were distributed and returned, 23 were excluded due to incorrect answers on lie-detection items, and a further 11 were removed because their response time fell outside the reasonable range (<100 or >1000 s). The analytic sample consisted of 1311 participants (97.5% response rate). All reported analyses are based on this sample. Participants comprised 440 males (33.6%) and 871 females (66.4%); the grade distribution included 437 (33.3%) in Grade 10, 319 (24.3%) in Grade 11, and 555 (42.3%) in Grade 12. All participants voluntarily signed a consent form prior to the start of the study. This indicates that they made the decision to participate only after fully understanding the research content and potential implications.

### 2.2. Research Instruments

#### 2.2.1. Mindfulness

Mindfulness was assessed using the 20-item Chinese short form of the Five Facet Mindfulness Questionnaire (FFMQ-20) revised by Zhong et al. (2016). This scale measures five facets: observing, describing, acting with awareness, non-judging, and non-reactivity, totaling 20 items. The authority and accuracy are widely recognized, demonstrating good reliability and validity in previous studies. Items 2, 4, 5, 6, 8, 13, and 20 were reverse-scored. Participants indicated their responses on a 5-point Likert scale (1 = “Strongly untrue of me”, 5 = “Strongly true of me”). On this scale, higher values correspond to more pronounced mindfulness traits. Sample items include “I am good at finding words to describe my feelings”; “I get easily distracted”; “I notice the smells and aromas of things.” In this study, the scale demonstrated an internal consistency reliability of Cronbach’s α = 0.641. Confirmatory factor analysis (CFA) was conducted to examine structural validity, with model fit evaluated against the following widely accepted criteria: χ^2^/df < 5, GFI > 0.90, CFI > 0.90, IFI > 0.90, and RMSEA < 0.08 (El-Hamamsy et al., 2024; Miao & Guo, 2025). The validity criteria for the following scales are identical. The CFA yielded the following model fit indices: χ^2^/df = 4.695, GFI = 0.953, CFI = 0.915, IFI = 0.915, RMSEA = 0.053.

#### 2.2.2. Future Time Perspective

The Future Time Perspective Scale developed by Q. Song (2004) was used. It includes five dimensions: future image, purpose awareness, future time efficacy, distant goal orientation, and behavioral commitment, totaling 20 items. Items 2, 17, 18, 19, and 20 were reverse-scored (see Appendix A). Participants’ responses were recorded on a 5-point Likert scale (1 = “Very untrue of me”, 5 = “Very true of me”). Higher scores indicate a stronger FTP. Sample items include: ”I have goals to strive for every day”; “I believe my future is mainly determined by fate”; “I often remind myself not to forget my most important future goals.” The internal consistency reliability for this scale was found to be Cronbach’s α = 0.914. The fit indices from the CFA indicated a well-fitting model: χ^2^/df = 4.524, GFI = 0.958, CFI = 0.962, IFI = 0.962, RMSEA = 0.052.

#### 2.2.3. Academic Positive Emotions

Two dimensions measuring high school students’ APE were selected from the Academic Emotions Questionnaire compiled by Dong and Yu (2007): the positive high-arousal academic emotions dimension (pride, pleasure, and hope) and positive low-arousal academic emotions dimension (satisfaction, calmness, and relaxation). The assessment comprises 10 items. Participants responded on a 5-point Likert scale (1 = “Strongly Disagree”, 5 = “Strongly Agree”). Elevated scores signify greater intensity of APE. Sample items are: “I feel proud that I solve problems faster than other classmates”; “Sometimes my learning mood is very high”; “I can study with peace of mind.” The scale demonstrated an internal consistency reliability of Cronbach’s α = 0.801 in this study. The CFA results indicated an excellent model fit: χ^2^/df = 3.192, GFI = 0.990, CFI = 0.992, IFI = 0.992, RMSEA = 0.041.

#### 2.2.4. Learning Burnout

The Learning Burnout Scale for Middle School Students developed by Hu and Dai (2007) was used. The scale covers four key dimensions: emotional exhaustion, alienation from teachers, physical depletion, and low learning efficacy. It consists of 21 items. Items 1, 5, 7, 11, 16, and 21 were reverse-scored. Responses were rated on a 5-point Likert scale (1 = “Strongly Disagree”, 5 = “Strongly Agree”). Higher scores indicate more severe levels of LB. Sample items include: “Studying brings me mental pleasure”; “For every exam, I have a feeling of just getting by”; “I do not trust what the teachers say.” The internal consistency reliability for this scale was found to be Cronbach’s α = 0.915. The CFA fit indices evidenced a good model fit: χ^2^/df = 3.713, GFI = 0.960, CFI = 0.956, IFI = 0.956, RMSEA = 0.046.

### 2.3. Procedure and Data Processing

The survey was administered by class. First, the instructions were read aloud to clarify the purpose of the test, answering method, and principles of voluntary participation and anonymity. Then, participants completed the questionnaire independently and honestly. Finally, questionnaires with incorrect lie-detection answers or excessively long or short completion times were excluded. We performed all statistical analyses with SPSS 21.0 and Amos for data processing and model testing. Categorical data were expressed as frequency and percentage (%). All data underwent normality tests and homogeneity of variance tests. Normally distributed continuous data are reported as mean ± standard deviation (*M* ± *SD*), while non-normally distributed data are presented as median and quartile [Q2(Q1, Q3)]. Except for gender, all variables in the model were standardized. The hypothesized serial mediation was tested using Model 6 from Hayes’s (2022) PROCESS macro for SPSS, with all variables in the analysis standardized except for the categorical demographics of gender. Statistical mediation was examined via bias-corrected bootstrapping (5000 iterations, 95% CI) within the PROCESS plugin (Hayes, 2022) for SPSS. An effect was considered significant if the 95% confidence interval (CI) did not include zero. Standardized coefficients were reported uniformly.

## 3. Results

### 3.1. Common Method Bias Test

To alleviate potential common method bias, procedural control measures were adopted during the data collection phase. These included the use of well-validated scales, incorporating reverse-scored items, and emphasizing the anonymity of this questionnaire. The assessment for common method bias, performed via Harman’s single-factor test, revealed 14 factors with eigenvalues > 1. The first factor explained 24.34% of the variance, which is below the 40% critical value, thus supporting the absence of severe common method bias (Zhou & Long, 2004). Furthermore, the collinearity diagnostics showed that the VIF values ranged from 1.01 to 1.87, all of which were below 10, ruling out concerns of multicollinearity in this study (H. Zhang et al., 2026).

### 3.2. Comparison of Variable Differences Across Demographic Factors

Difference tests were conducted on the scores of each variable across different levels of gender and grade. The findings revealed a statistically salient gender difference in mindfulness. However, no significant gender differences were found in LB, FTP, or APE. Specifically, male students attained significantly higher scores on mindfulness than female students. No statistically significant differences were detected in the variable comparisons across grade levels. The detailed findings are summarized in Table 1.

### 3.3. Descriptive Statistics and Correlation Analysis

Partial correlation analysis, controlling for gender, indicated that mindfulness was significantly negatively correlated with LB and significantly positively associated with FTP and APE. LB was significantly negatively associated with FTP and APE, while FTP was significantly positively associated with APE. Table 2 displays the correlation matrix, along with the key variables’ mean (*M*) and standard deviation (*SD*).

### 3.4. Chain-Mediation Effect Analysis

**Figure 2:**
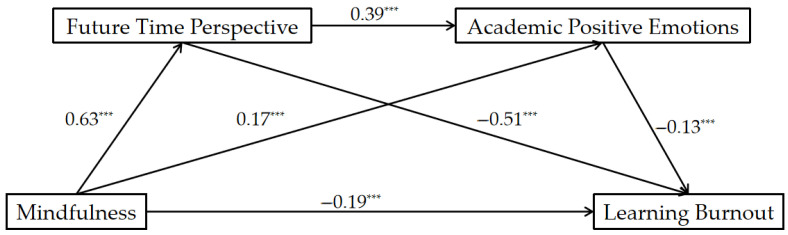
Chain-Mediation Model. *** *p* < 0.001 (two-tailed).

After controlling for gender, we conducted linear regression and chain-mediation analyses. In these analyses, mindfulness level served as the predictor, LB as the outcome, and FTP and APE as mediators. The outcomes were as follows: The chain-mediation model was significant and the total effect of the model was −0.56. First, the direct effect of mindfulness → LB proved significant, exhibiting a direct effect of −0.19 and representing 33.93% of the total effect, thus supporting Hypothesis 1. Second, indirect effect 1 via mindfulness → FTP → LB was significant, recording an indirect effect of −0.32 and making up 86.48% of the overall indirect effect, supporting Hypothesis 2. Third, the mindfulness → APE → LB indirect path was substantiated (estimate = −0.02), representing 5.41% of total indirect effect, supporting Hypothesis 3. Finally, the mindfulness → FTP → APE → LB chain mediation was significant (estimate = −0.03), amounting to 8.11% of overall indirect effect, affirming Hypothesis 4. Collectively, the overall indirect effect amounted to −0.37, representing 66.07% of the total effect. We employed hierarchical regression to assess the incremental contribution of the mediators. After controlling for gender, we compared a baseline model (mindfulness only) against a sequential mediation model including FTP and APE. The mediators jointly accounted for an additional 20% of the variance in LB, Δ*R*^2^ = 0.20, *F*(2, 1306) = 274.36, *p* < 0.001. Mindfulness indirectly was associated with LB through independent mediating effects and the chain-mediating effect of FTP and APE. A schematic of the theoretical model is provided in Figure 2. Readers are referred to Table 3 for a summary of the comprehensive regression analysis.

We employed bias-corrected percentile bootstrap to evaluate the model’s effects. The results indicated that none of the Bootstrap 95% CIs for the three indirect effects included 0, confirming their statistical significance. This confirms the existence of a chain-mediating effect of FTP and APE in the relationship between mindfulness and LB, supporting the hypothesized model. Detailed results are shown in Table 4.

## 4. Discussion

Grounded in self-regulated learning model, this research incorporated cognitive and motivational lenses to analyze the relationship between mindfulness and LB. The analyses converged to show that mindfulness directly and inversely predicted LB, while also exerting its influence through a significant serial mediation of FTP and APE.

This study confirms the direct association between mindfulness and LB among high school students. According to the Self-Determination Theory, mindfulness, as an important internal personal resource, helps individuals focus their attention on meeting current demands, enhancing their sense of control, and satisfying their needs for autonomy and competence (Y. Li et al., 2021). It can also facilitate individuals adopt more adaptive coping strategies when confronted with events (Y. Chen et al., 2023), thereby reducing their likelihood of developing LB. In specific academic contexts, mindfulness in high school students can boost confidence in their own abilities, compensate for the unmet psychological needs caused by LB, prevent the resulting “efficacy crisis,” and reduce LB. This is consistent with previous findings, demonstrating that mindfulness inversely predicts LB at a significant level (M. Jin et al., 2022; Y. Zhang & Wang, 2024). Meanwhile, by enhancing an individual’s awareness of present experiences, capacity for concentration, and emotion regulation ability, mindfulness can amplify feelings of immersion and pleasure. This, in turn, facilitates psychological recovery and relaxation, ultimately alleviating LB. Additionally, it cultivates an open and accepting stance toward experiences, enabling individuals to recognize and regulate maladaptive cognitions and emotions in a more automatic and immediate manner (Creswell, 2017), which helps mitigate LB.

The present research verified that mindfulness robustly predicts LB through FTP mediation. Derived from the balanced time perspective theory (Rönnlund et al., 2019), individuals with high mindfulness levels possess a more positive present-time perspective, which enhances the positivity of their future-time perspective, that is, it strengthen their FTP. Furthermore, according to the Goal Setting Theory, FTP assists individuals in identifying the practical value of current behaviors and future repercussions, leading to clearer and more determined learning goals, more stable and persistent goal-directed behavior, richer adaptive behaviors, and consequently reduced LB (Pang et al., 2014). This mediating process is partially supported by empirical research. Previous studies have shown that mindfulness can significantly positively predict FTP (Kabat-Zinn, 2003), which directly associations LB (F. Zhang et al., 2016). In summary, mindfulness can effectively help students focus on the present, enhance their FTP, prompt them to adopt more positive coping strategies to facilitate psychological regulation (Y. Chen et al., 2023), and reduce LB.

This study confirmed that mindfulness was significantly associated with LB through the mediating role of APE. The findings substantiate key propositions of the BBT of positive emotions and academic emotions theory. These findings align with prior research, which suggests mindfulness training fosters positive emotional experiences and markedly alleviates LB (Kinnunen et al., 2020). Mindfulness helps individuals focus attention on behavioral goals, reducing sensitivity to negative emotional stimuli, and increasing tolerance and acceptance of adverse environments (Ganaden & Smith, 2010; Zeidan et al., 2010; Jha et al., 2010; Schuman-Olivier et al., 2020). It also enhances individuals’ conscious sensitivity to negative emotions, making them less likely to be overwhelmed by negativity, enabling timely adaptive cognitive choices and decisions, thereby helping them pursue positive emotional states (Erisman & Roemer, 2010). This allows for a deeper experience of pleasure from positive life events and amplifies their positive effects (Brown & Cordon, 2009). Simultaneously, APE can directly reduce individuals’ LB and indirectly diminish LB by expanding immediate thought-action repertoires, building enduring personal resources, and reducing emotional exhaustion (Fredrickson, 2001). Therefore, mindfulness is a viable approach to managing academic emotions to improve attentional function, nurture constructive academic emotions, including pride, satisfaction, and hope, and reduce LB.

This study found that the chain-mediating effect of FTP and APE plays a role in the association between mindfulness and LB. This confirms that the cognitive and motivational structures of the self-regulated learning model function through a cognitive-motivational progressive mechanism, providing empirical evidence for the model and strongly corroborating it (Edisherashvili et al., 2022). Those with a strong capacity for mindfulness levels maintain a sharper focus on current events and states, possess stronger FTPs, experience richer and more frequent APE during learning, exhibit stronger learning motivation and greater learning engagement, and are less likely to experience LB. This overcomes the limitations of previous research, which predominantly used emotional-affective variables as mediators (Currie, 2020; Zhu & Chang, 2021).

This study explored the pathways linking mindfulness to high school students’ LB from cognitive and motivational perspectives through a chain-mediation model of FTP and APE. It enriches the self-regulated learning model and provides strong evidence for the sequential roles of meta-cognitive, cognitive, and motivational-affective components in influencing learning behaviors, while also offering insights into theoretical models of mindfulness and prompting reflection on their mechanisms in complex cognitive processes.

Furthermore, this study provides a new approach to addressing LB among high school students, contributing to the enhancement of their academic well-being, the improvement of their learning experiences and mental health, and the promotion of their holistic development. It provides practical guidance for educators in practical teaching activities. Specifically, they can flexibly integrate professional mindfulness training into classroom design, increasing classroom engagement while helping high school students focus on the present class, relieving academic pressure, and reducing LB. Concurrently, it offers insights for them to mitigate students’ psychological stress through mindfulness practices, cultivate their FTP, integrate a positive future time orientation into daily teaching, enrich their experience of APE, and ultimately alleviate LB.

The present research is not without limitations. First, the data were derived exclusively from participants’ self-reports, which may be subject to errors such as social desirability bias and memory bias. To address these limitations, future studies could consider several refined approaches: (1) incorporate established social desirability scales directly into the questionnaire (e.g., Reynolds’s (1982) short form of the Marlowe-Crowne Scale) for direct measurement and control; (2) employ indirect measurement methods like the “best friend” projection technique to reduce response distortion on sensitive topics (Rojas-Méndez & Davies, 2024); (3) adopt multi-paradigm assessment methods from clinical or experimental psychology to mitigate memory bias (Schacter et al., 2011); and (4) expand data sources by incorporating reports from peers, parents, or teachers to obtain more objective and complementary information. Secondarily, all participants were recruited from a single high school, which may limit the generalizability of the findings to students from different types of schools or educational stages. Future research should expand the sampling strategy to include a more diverse population and explore how school-level characteristic variables might influence the model. Finally, the present study was cross-sectional in nature, implemented through survey methodology. Although the analysis and discussion of the hypothesized model have a solid research foundation, causal relationships between variables and their long-term effects cannot be definitively established. Future studies should employ longitudinal or experimental designs to further verify the temporal sequence and underlying causal mechanisms among these variables.

## 5. Conclusions

In our model, mindfulness appeared to significantly affected LB negatively among high school students and indirectly was associated with LB through independent mediating effects and the chain-mediating effect of FTP and APE.

This study provides strong evidence for the sequential roles of meta-cognitive, cognitive, and motivational-affective components in the self-regulated learning model. Moreover, it offers practical guidance for educators, suggesting that mindfulness can alleviate psychological stress, enhance FTP, foster APE, and mitigate LB.

## Figures and Tables

**Table 1:** Differences in variable scores by demographic factors (*M* ± *SD*).

Variable	Type	Mindfulness	Learning Burnout	Future Time Perspective	Academic Positive Emotions
Gender	Male (*n* = 440)	58.73 ± 6.10	54.51 ± 12.59	68.81 ± 11.24	33.16 ± 5.46
	Female (*n* = 871)	57.74 ± 6.46	54.87 ± 11.52	68.18 ± 10.59	32.58 ± 5.11
	*t*	2.68	−0.50	1.00	1.89
	*p*	0.007	0.617	0.319	0.059
	Hedges’s g	0.157	0.030	0.058	0.110
Grade	Grade 1 (*n* = 437)	58.34 ± 6.57	53.64 ± 11.98	68.32 ± 10.90	32.69 ± 5.15
	Grade 2 (*n* = 319)	57.76 ± 6.29	55.12 ± 12.01	67.45 ± 10.79	32.58 ± 5.37
	Grade 3 (*n* = 555)	58.04 ± 6.23	55.40 ± 11.69	68.98 ± 10.74	32.96 ± 5.22
	*F*	0.76	2.92	2.04	0.64
	*p*	0.469	0.054	0.130	0.528
	*η_p_^2^*	0.001	0.004	0.003	0.001

We computed Hedges’ g (adjusted for small-sample bias) as the effect size for all *t*-tests and generated 95% confidence intervals.

**Table 2:** Results of correlation analysis among main variables.

Variable	*M*	*SD*	1	2	3	4
1 Mindfulness	58.07	6.36	1			
2 Learning Burnout	54.75	11.88	−0.57 ***	1		
3 Future Time Perspective	68.39	10.81	0.63 ***	−0.69 ***	1	
4 Academic Positive Emotions	32.78	5.23	0.41 ***	−0.46 ***	0.50 ***	1

*** *p* < 0.001 (two-tailed).

**Table 3:** Regression analysis for chain-mediation.

Regression Equation		Overall Fit Indices				Significance of Regression Coefficients			
Dependent Variable	Independent Variable	*R*	Adjusted *R^2^*	∆*R^2^*	*F*	*β*	Bootstrap *LL*	Bootstrap *UL*	*t*
LB	M	0.57	0.32	0.32	308.14 ***	−0.57	−0.60	−0.51	−24.82 ***
LB	M	0.71	0.51	0.19	452.75 ***	−0.21	−0.26	−0.16	−8.52 ***
	FTP					−0.56	−0.59	−0.50	−22.47 ***
LB	M	0.62	0.38	0.06	269.29 ***	−0.45	−0.49	−0.40	−18.97 ***
	APE					−0.27	−0.31	−0.22	−11.43 ***
LB	M	0.72	0.52	0.20	355.65 ***	−0.19	−0.24	−0.14	−7.69 ***
	FTP					−0.51	−0.55	−0.45	−19.50 ***
	APE					−0.13	−0.17	−0.08	−5.66 ***
APE	M	0.42	0.17	0.17	136.95 ***	0.41	0.37	0.47	16.42 ***
APE	M	0.52	0.27	0.09	158.45 ***	0.17	0.11	0.23	5.39 ***
	FTP					0.39	0.34	0.46	12.91 ***
FTP	M	0.63	0.40	0.40	431.80 ***	0.63	0.59	0.68	29.36 ***

All continuous variables in the model have been standardized. M stands for Mindfulness, FTP for Future Time Perspective, APE for Academic Positive Emotions, and LB for Learning Burnout. *** *p* < 0.001 (two-tailed).

**Table 4:** Analysis of the mediating effects of FTP and APE.

Effect	Effect Value	Effect Proportion(%)	Boot *SE*	Boot *LL CI*	Boot *UL CI*
Indirect Effect 1	−0.32	57.14	0.02	−0.36	−0.27
Indirect Effect 2	−0.02	3.57	0.01	−0.03	−0.01
Indirect Effect 3	−0.03	5.36	0.01	−0.05	−0.02
Total Indirect	−0.37	66.07	0.02	−0.41	−0.32
Direct Effect	−0.19	33.93	0.02	−0.24	−0.14
Total Effect	−0.56	100	0.02	−0.60	−0.51

## Data Availability

The raw data supporting the conclusions of this article will be made available by the authors upon request.

## Appendix A. The Future Time Perspective Scale

The following questions are designed to reflect your perception of time. Please indicate how well each statement describes your actual behavior over the past month. Use the scale below, where 1 represents “Very untrue of me,” 2 represents “Somewhat untrue of me,” 3 represents “Uncertain,” 4 represents “Somewhat true of me,” and 5 represents “Very true of me.” Please read each item carefully and check (√) the number that best corresponds to your situation.

**Item**	**Very Untrue**	**Somewhat Untrue**	**Uncertain**	**Somewhat True**	**Very True**
1. I have goals to strive for every day.	1	2	3	4	5
2. I believe my future is mainly determined by fate.	1	2	3	4	5
3. I often remind myself not to forget my most important future goals.	1	2	3	4	5
4. I believe I have the ability to build a bright future for myself.	1	2	3	4	5
5. Looking ahead, there are many things I need to accomplish.	1	2	3	4	5
6. I am aware of what my main tasks are at present.	1	2	3	4	5
7. Once I set a goal, I take concrete steps to achieve it.	1	2	3	4	5
8. I often envision the goals I want to achieve in five years.	1	2	3	4	5
9. I believe my future will be wonderful.	1	2	3	4	5
10. I evaluate information related to life development based on whether it helps me achieve my long-term goals.	1	2	3	4	5
11. I often imagine how I will change throughout my future life course.	1	2	3	4	5
12. Once I decide what to do, I consider how to accomplish it.	1	2	3	4	5
13. I know there are many tasks to complete in the future.	1	2	3	4	5
14. I complete my plans on time by making steady progress.	1	2	3	4	5
15. I often reflect on what my long-term life goals are.	1	2	3	4	5
16. I am full of confidence about my future.	1	2	3	4	5
17. The course of my life is determined by forces beyond my control.	1	2	3	4	5
18. I often feel that life has no purpose.	1	2	3	4	5
19. I pay considerable attention to negative evaluations from others about my future development.	1	2	3	4	5
20. My understanding of my own future is very vague.	1	2	3	4	5
